# Biochemical Characterization of Traditional Varieties of Apricots (*Prunus armeniaca* L.) of the Campania Region, Southern Italy

**DOI:** 10.3390/foods11010100

**Published:** 2021-12-31

**Authors:** Florinda Fratianni, Antonio d’Acierno, Donatella Albanese, Marisa Di Matteo, Raffaele Coppola, Filomena Nazzaro

**Affiliations:** 1 Institute of Food Science, CNR-ISA, Via Roma 64, 83100 Avellino, Italy; florinda.fratianni@isa.cnr.it (F.F.); coppola@unimol.it (R.C.); filomena.nazzaro@isa.cnr.it (F.N.); 2 Department of Industrial Engineering, University of Salerno, Via Giovanni Paolo II, 84084 Fisciano, Italy; dalbanese@unisa.it (D.A.); mdimatteo@unisa.it (M.D.M.); 3 Department of Agriculture, Environment and Food, University of Molise, Via de Sanctis Snc, 86100 Campobasso, Italy

**Keywords:** apricot, polyphenols, antioxidant, biodiversity

## Abstract

Campania is the most important region of Italy in the apricot cultivation, present mostly in the Vesuvio area. At least to the best of our knowledge, no studies are reporting the biochemical characterization of the considerable number of traditional apricot varieties present on this territory, including the qualitative and quantitative profile of the polyphenols present. Our work evaluated the content of β-carotene, total phenolics, phenolic profiles, ascorbic acid and antioxidant activity of 23 traditional varieties of apricots of the Campania region. Principal component analysis (PCA) highlighted that, in the two main groups, the antioxidant activity was strongly affected by the content of ascorbic acid (−0.89), or slightly affected by the content of total polyphenols (−0.67), respect to the content of ascorbic acid (−0.55), never by β-carotene. Chlorogenic acid (up to 55.07 μg g^−1^) and catechin (up to 96.15 μg g^−1^) resulted the most abundant polyphenols recognized through the chromatographic analysis. PCA, extended to the polyphenol profile, confirmed the distribution of the varieties in two large groups, evidencing once again the hierarchical distance of four varieties (“Panzona”, “Paolona” “Baracca” and “Boccucia Eboli”) compared to the others.

## 1. Introduction

Apricot (*Prunus armeniaca* L.) is one of the most important fruits produced in temperate countries. The typical apricot grows in geographically diverse areas ranging from the Siberia cold winters to North Africa, characterized by a subtropical climate of North Africa and from the desert Central Asia to Japan and Eastern China [1]. Its total world production moves to about 2.6 million tons. Uzbekistan and Turkey are the leading countries in the production of apricot fresh fruits [www.fao.org/faostat/en/#data/QC. accessed on 10 November 2021] and mainly Turkey, for the manufacturing of dried apricots [2]. Italy is the fifth worldwide and the first European producer of apricots, with 222,690 tons [www.fao.org/faostat/en/#data/QC. accessed on 10 November 2021]. In Italy, the production of apricots is concentrated primarily in Campania, and Emilia-Romagna [3]. Campania is the most important region of Italy in the apricot cultivation, with about 50,000 tons of product, present mainly in the Vesuvio area, which represents around 80% of the regional production. In this area, there are about 2000 hectares of apricots field, with a production that, in normal climate conditions, should be settled around 400,000 quintals, mostly consumed as fresh fruit.

A certain portion-variable from year to year- is transformed into nectars, i.e., juice and pulp, while a small part is transformed to give jams, dried and candied, and ultimately a minimal portion is transformed into frozen, and canned products (http://agricoltura.regione.campania.it/tipici/albicocca.htm. accessed on 7 July 2021). Climate conditions and variety influence the growth and ripening of the fruit. The fruit has the shape of a drupe, similar to a plum, with a thin outer skin walling the yellow flesh. It has several shades of color ranging from yellow to orange with a reddish random overlay, seen as parameters affecting its quality [4,5]. Apricot-consumed both as fresh and dried- is thus an important component of the human diet. Its main phytochemicals possess well-known biological properties, comprising antioxidant [6,7], anti-microbial [8], and anti-inflammatory [9].

The importance of apricot and by products for the human health is well ascertained. In fact, it can exert different positive effects against such as cancer, cardiovascular disease, atherosclerosis and those diseases related to the aging. Furthermore, it can have protective effect on kidney and liver [10]. 

Apricot contains several secondary metabolites [11,12,13], many of them being active as antioxidants [14]. Polyphenols and carotenoids (mainly β-carotene) represent the most abundant classes of phytochemicals contained in this fruit [15]; such molecules are not only crucial for the antioxidant activity but play a primary role also in the color and taste in fruit [4]. In addition, apricots contain a substantial amount of ascorbic acid. Due to the wide range of existing varieties, identifying the best genotypes assumes particular importance for both breeders and consumers, which can select and consume those specific products with high nutritional quality, respectively. Therefore, the present study focused on the following aims:

To analyze the content of β-carotene, total phenolics, phenolic profiles, ascorbic acid of different traditional landraces of apricots of the Campania region, Southern Italy.

To determine the in vitro antioxidant activity by the DPPH radical-scavenging activity.

To correlate the antioxidant activity of the landraces to total phenolics, ascorbic acid, and carotenoids aimed to identify the principal factors affecting the antioxidant properties of the products.

Principal component analysis (PCA) investigated the interrelationships between these parameters and the different varieties. 

## 2. Materials and Methods

### 2.1. Chemicals

The Folin–Ciocalteu reagent was purchased from BIO-RAD (Milano, Italy). Chlorogenic, gallic, caffeic, ferulic, *p*-coumaric acids, epicatechin, rutin, quercetin, 2,2-diphenyl-1-picrylhydrazyl (DPPH), ascorbic acid, β-carotene, HPLC-grade methanol, metaphosphoric, sulphuric, formic and acetic and acids, acetonitrile, petroleum ether, ethanol and acetone were acquired from Sigma-Aldrich (Milano, Italy). Apigenin was bought from Extrasynthese (Genay, France). Distilled water was obtained from a Milli-Q apparatus (Millipore, Milano, Italy).

### 2.2. Plant Material

Plant material included 23 varieties of apricot (*Prunus armeniaca* L.), “Baracca”; “Boccuccia Eboli”; “Boccuccia Grossa”; “Boccuccia Liscia”;“Campana”; “Diavola”; “Fracasso”; “Fronne Fresche”; “Lisandrina”; “Magnolona”; “Montedoro”; “Nonno”; “Panzona”; “Paolona”; “Pazza”; “Puzo”; “San Francesco”; “Scassulillo”; “Scassulillo Grande”;“Signora”; “Zeppona”; “Pollastrella”; “Boccuccia Spinosa” listed by the Official Bulletin of the Campania Region (B.U.R.C. n. 42, 145, 2009), grown and collected during the month of June 2011 in the experimental plant “Improsta” located in Eboli (SA), of the Campania region, Italy (40.5569° N, 14.9832° E), characterized by a Mediterranean climate by an average of air temperature (T) = 23.9 °C, humidity (U) = 64.9%, wind (W) = 8.1 km/h, rainy days (R) = 6 (https://www.ilmeteo.it/portale/archivio-meteo/Eboli/2011/Giugno. accessed on 10 November 2021) during the growing season. 

As control we used another variety of apricot: “Tipo 35”. Fruits were harvested at the physiological maturity (ready-to-eat), visually when the fruits were 80% yellow color on the surface, and immediately transported to the laboratory. Herein, 0.5 kg for each variety were well mixed to obtain three independent replicates and stored at −26 °C before the analysis. All the analysis were made in triplicate.

### 2.3. Dosage of Ascorbic Acid

Samples were cut, pressed, and kept into the dark for 1 h at 4 °C in three volumes of metaphosphoric acid (4%) following the method of Nazzaro et al. [16]. Then, they were centrifuged (11,600× *g* for 10 min at 4 °C, Biofuge, Beckman Italia, Cassina de’ Pecchi, Milano, Italy); the supernatant was collected after filtration (0.45 μm mesh, Millipore, Milano, Italy). The dosage of ascorbic acid was performed by HPLC-UV (Gold System, Beckman Italia, Cascina dè Pecchi, MI, Italy) using a Khromasil KR 100-5 C18 column (25 cm × 4.6 mm). The run was performed at room temperature, at the following analytical conditions: mobile phase = HPLC-grade water acidified with sulphuric acid 0.001 M; flow rate: 1.0 mL min^−1^; detection wavelength = 245 nm; injection volume = 20 μL. The standard curve was obtained using ascorbic acid dispersed in the mobile phase. Results were indicated as mg 100 g^−1^ of fresh sample.

### 2.4. Carotene Content

Samples were cut and pressed in ethanol (1:1 *w/v*); subsequently we included petroleum ether (1.5:1 *v*/*v*) [17]. Mix was robustly agitated and centrifuged (11,600× *g*, 15 min; Biofuge, Beckman Italia), then we recuperated the supernatant. The steps were repeated up until the absolute loss of the color, and the supernatants were put jointly. The amount of carotenoids was assessed at λ: 450 nm (Cary 50 Uv/Vis Varian-Agilent Italia, Cernusco sul Naviglio, Italy) utilizing petroleum ether as blank, and considering ε = 2592 as the extinction coefficient. Results were indicated as mg of β-carotene 100 g^−1^ of fresh sample.

### 2.5. Total Polyphenols

Samples were cut and pressed (1:3 *w/v*) in acidified methanol (acetic acid 1%); subsequently, they were kept overnight at 4 °C [18]. Supernatants were collected after centrifugation (11,600× *g*, 15 min; Biofuge, Beckman Italia). The total polyphenols content was assessed (Cary 50 Varian-Agilent) at λ = 760 nm, following the method of Singleton and Rossi [19]. Gallic acid was used to generate the standard curve. Results were expressed in terms of mg gallic acid equivalent 100 g^−1^ of fresh sample. 

### 2.6. Antioxidant Activity

Samples were cut and pressed (1:3 *w/v*) in an acidized solution of methanol (containing acetic acid 1%); then, they were kept overnight at 4 °C ± [18]. Supernatants were recovered by centrifugation (11,600× *g*, 15 min; Biofuge, Beckman). The stable radical 2,2-diphenyl-1-picrylhydrazyl (DPPH assay) was used to evaluate the radical-scavenging activity of our samples [20]. The analysis was achieved in microplates. 15 μL of extract were added to 300 μL of a solution of methanol-DPPH (6 × 10^−5^ M). The absorbance was measured at λ = 517 nm (Cary 50 MPR Varian-Agilent). The EC50 indicated the amount of sample amount (as mg) required to inhibit, after 60 min of incubation, the activity of 1 mL of the DPPH by 50%. 

### 2.7. Chromatographic Analysis

Polyphenol profile of the different cultivars of apricot was evaluated by ultra-high-performance liquid chromatography (UPLC) analysis using the ACQUITY Ultra Performance LC^TM^ system (Waters, Milford, MA, USA) connected to a PDA 2996 photodiode array detector (Waters). The connected Empower software (Waters) allowed the control of the instruments and the acquisition and processing of the relative data. The analysis was performed following the methods described by Pane et al. [21]. Extracts and standards were dispersed in methanol; then they were filtered using microfilter units Whatman 0.45 μm (Waters, Milford, MA, USA). Running conditions = Injection volume: 5 μL. Mobile phase: solvent A (7.5 mMol acetic acid) and solvent B (acetonitrile); flow rate: 250 μL min^−1^; column: reversed-phase column (BEH C18, 1.7 μm, 2.1 × 100 mm Waters); temperature: 30 °C. Each analysis was performed with a gradient elution (0.8 min: 5% B for; 5.2 min: from 5% to 20% B; 0.5 min: 20% B; 1 min: from 20 to 30% B; 0.2 min: 30% B; 2.3 min: from 30% to 50% B; 1 min: from 50% to 100% B; 1 min:100% B; 0.5 min to reach 5% B from 100% B. Then, the column was restored to the initial conditions for 2.5 min. Quantification of polyphenols were made based on linear curves of the standards.

### 2.8. Statistical Analysis

Data were expressed as mean ± standard deviation of triplicate measurements. Calculations were performed through the PC software “Excel Statistics”. Principal component analysis (PCA) was used to evaluate the interrelationships between the biochemical parameters and the different varieties, following Fratianni et al. [19] through the software package MATLAB.

## 3. Results and Discussion 

Apricot is source of several important secondary metabolites, in particular polyphenols, β-carotene and ascorbic acid. Thus, this fruit is also font of antioxidants. We have determined the content of such metabolites, and the antioxidant activity, of different traditional varieties of apricots present in the territory of the Campania region. The content of ascorbic acid, b-carotene and total polyphenols was expressed as mg 100 of fresh weight, following Wani et al. (2017) [22]. Results are shown in Table 1.

### 3.1. Ascorbic Acid Content

The quantity of ascorbic acid found in the apricot varieties analyzed varied between 3.47 mg (“Signora”) and 10.08 mg in 100 gr of the fresh product (“Magnolona”). Some of them (“Magnolona”, “Scassulillo grande “, “Boccuccia spinosa”, “Fracasso”) presented content of ascorbic acid close to those exhibited by other varieties analyzed by Ishaq et al. [23]. Many of them contained an amount of ascorbic acid superior to that present in the apricot “tipo 35” used as control; for some of them, the content of ascorbic acid was double (“Scassulillo” “Lisandrina”) or more than double (“Boccuccia Spinosa”, “Fracasso”, “Magnolona”, and “Scassulillo grande”) compared with that showed the control. All the varieties had a vitamin C content similar to that found by Hegedus et al. [24], but inferior, although in some varieties, such as “Magnolona”, “Scassulillo grande” and “Fracasso” just slightly less, than those reported by Akin et al. [25], which, in 11 apricot varieties of Malatya (Turkey), known as “the city of apricots”, found a vitamin C content of 11.5 mg 100 g^−1^, if calculated based on the fresh weight. The presence of ascorbic acid is an essential factor not only for the beneficial effects that such molecule can exert on health but also for applications relating to the food industry, since it is attributed to the ascorbic acid a role in inhibiting the action of polyphenol oxidases and therefore in delaying the browning of the fruits [26]. The amount of ascorbic acid was higher than those reported by Cui et al. 2019 [27], and, for the majority of the cultivars analyzed by us, greater than those reported by Wani et al. [28], and Kafkaledou [29], which observed in eight apricot cultivars a content never higher than 0.987 mg 100 g^−1^ of product. Our data resulted in higher also respect to those indicated by Nourozi and Sayyari [30], about the variety Nouri that never exceeded 2.4 mg kg^−1^ of fresh product. Within the cultivars we analyzed, it is to highlight the different behavior exhibited by the 4 varieties “Boccuccia”, which content of ascorbic acid ranged between 4.09 mg mg 100 g^−1^ of fresh product (“Boccuccia grossa”) to more the double (9.80 mg/100 g of the fresh product, variety “Boccuccia spinosa”). The significant difference in ascorbic acid, as well as that reported for the other parameters evaluated (see below) among the apricot varieties observed in our study, in this case could be mostly attributed to the influences of factors such genotypic differences more than the year of collection and the period of maturity [22].

### 3.2. β-Carotene Content

For some years, the nutritional importance of carotenoids has been related to their capacity to act as a precursor of vitamin A, retinol. Such a peculiarity is essential and is typical of β-carotene, greatly helpful for our body, to supply a quantity of 1 mg, we need 6 mg of β-carotene, on the other hand, for all other carotenoids, and such rate is 12:1 (https://www.inran.it/carotenoidi/8708/ accessed on 10 September 2021). The content of β-carotene found in our samples was very variable depending on variety (Table 1). Some of them exhibited an amount of β-carotene (“Puzo”, “Montedoro” and “Lisandrina”, “Fracasso”, “Fronne”,” Nonno” and “San Francesco”) not exceeding 0.1 mg 100 g^−1^ of fresh weight; other varieties showed a content of β-carotene never lower than 0.2 mg 100 g^−1^ of fresh weight. Three of them, “Baracca”, Panzona, and the “Boccuccia Eboli” showed no less than 0.3 mg 100 g^−1^ of fresh weight; the variety “Baracca” variety arrived to exceed 0.4 mg 100 g^−1^ of fresh weight, and resulted perfectly in line with the values reported by the Italian Council for the research in agriculture and analysis of agricultural economy (http://nut.entecra.it/646/tabelle_di_composizione_degli_alimenti.html?idalimento=007000 accessed on 5 November 2021). In addition, the four varieties “Boccuccia” (“Eboli”, “Grossa”, “Liscia”, and “Spinosa”) showed different results, with “Boccuccia spinosa” exhibiting the lowest amount of β-carotene (0.134 mg 100 g^−1^ of fresh weight), less than half if compared with the variety “Boccuccia Eboli”, which had 0.307 mg 100 g^−1^ of fresh weight. Such values were lower than those reported by Leccese et al. [31] on different Italian varieties of apricot, including “Boccuccia spinosa”, even if such varieties were cultivated in a different territory and probably collected in 2010, so, with diverse geographic, soil and climate situation. In any case, some of the varieties analyzed, such as “Paolona”,” Panzona”, three among the four varieties “Boccuccia” (“Eboli”, “Grossa” and “Liscia”), and variety “Signora” possessed a content of β-carotene undoubtedly superior to the apricot “tipo 35” used as control. Some of the varieties we analyzed showed a level of β-carotene superior to those grown in China and characterized by Zhou et al. [32], and some Moroccan varieties analyzed by Ayour et al. [33]. This could indicate that the amount of β-carotene could depend not only on the variety but also on the cultivation area (with variables including climate) and the year of cultivation. 

### 3.3. Total Polyphenols Content

Polyphenols constitute one of the principal founts of antioxidant activity. The quality and nutritional features of fruit and vegetables are affected by several parameters, such as the environment, and the cultivation practices [34]. Climate, light intensity, temperature, and the water accessibility can influence their antioxidant activity too, as well as their polyphenol content [35,36]. Phenolic compounds are vital for the plant tissue, as they are involved in the plant defense mechanism, through the prevention of pathogen growth, also by strengthening the vegetal tissues [37]. From a biological point of view, one of their most important roles is their antioxidant activity [38]. The amount of total polyphenols (TPs) also appeared very variable among the varieties analyzed. Many of them had a TPs content higher than that exhibited by the “tipo 35” that we used as a control. In some cases, the TPs content was also double (“San Francesco”), 2.17 times higher (“Baracca”) and it even reached 2.5 times greater (“Boccuccia Eboli”) than the control. Some varieties, such as “Fronne Fresche”, “Panzona”, “Pollastrella”, and “Scassulillo” had a TPs content of less than 10 mg. 100 g^−1^ of fresh weight. Furthermore, about total polyphenols, the group of varieties “Boccuccia” showed a not completely uniform behavior, with three of the varieties (“Boccuccia Grossa”, “Boccuccia Liscia” and “Boccuccia spinosa”) which contained a practically similar quantity of TPs. The exception was represented by the “Boccuccia Eboli” which, with 35.875 mg 100 g^−1^ of fresh weight, was also the variety with the highest TPs content. Our data resulted superior to those obtained by Nourazi et al. [30] on the Iranian variety “Nouri”, whose TP content did not exceed about 4.3 mg 100 g^−1^ of fresh weight. The content of total polyphenols exhibited by the variety “Boccuccia Eboli” was very similar to that contained in the variety “Neraida” [29], and in the variety Nostos [39]. Therefore, the content of total polyphenols of the variety “Baracca” was similar to the variety “Nereis” [39]. 

### 3.4. Antioxidant Activity

The antioxidant activity exhibited by the apricot varieties was calculated by means of the DPPH test, and expressed in terms of EC50, indicating the quantity, in mg, of the extract, necessary to inhibit the activity of 1 mL of the DPPH radical by 50% The data are shown in Table 1. In general, we observed that, except some varieties, in particular “Pollastrella” (EC50 = 13.49 mg), “Montedoro” (EC50 = 14.87 mg), and “Panzona” (EC50 = 17.14 mg)—this last exhibiting the weakest antioxidant activity—the other varieties showed EC50 values never exceeding 13 mg, and, for many of them, it was fewer than 9.0 mg. “Zeppona” (EC50 = 7.62 mg), “Campana” (EC50 = 7.46 mg), but above all “San Francesco” (EC50 = 6.88 mg) and “Boccuccia Eboli” (6.60 mg), exhibited the best antioxidant performances. All the varieties possessed antioxidant activity higher than that of “Halman” one, which EC50 was of about 46.6 mg/mL DPPH [40], or, considering the difference between fresh and dried product, also respect to that showed by commercial dried apricot analyzed by Canadanovic-Brunet et al. [41], or a commercial apricot bought in a local market in Algeria [42]. The capacity of apricot to exert a good antioxidant activity is of particular relevance. Apricots can ameliorate the inflammatory cell infiltration, epithelial desquamation, and microvillar damage occurring in the intestines of rats [43], and, from tests performed on rats, they could show a preventive effect, if regularly consumed, on oxidative stress due to ethanol, also limiting the subsequent histopathological changes [44].

**Figure 1:**
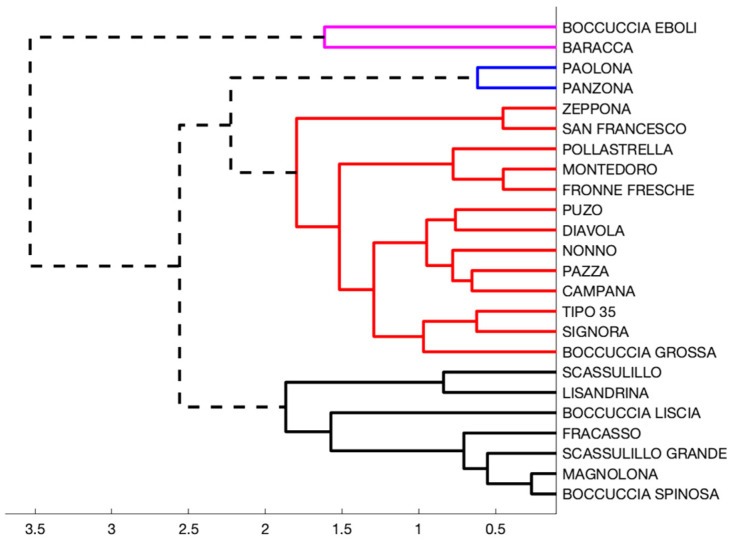
Hierarchical clustering of varieties obtained using a Euclidean unweighted distance and considering the normalized content of β-carotene, total polyphenols normalized content, and ascorbic acid normalized content. We used 1.9 as the cutoff distance.

Using the unweighted average Euclidean distance, we have hierarchically clustered data considering the normalized content of β-carotene, total polyphenols normalized content, and ascorbic acid normalized content. Results are shown in Figure 1.

We could identify four groups considering 1.9 as cutoff distance (1.9 is approximatively equal to half of the largest distance between clusters). For the most populated groups (the black and the red ones), we then analyzed the correlation among the antioxidant activity, the ascorbic acid content, and the total polyphenols content. Varieties in the black group (“Scassulillo”, “Lisandrina”, “Boccuccia Liscia”, “Fracasso”, “Scassulillo Grande”, “Magnolona”, and “Boccuccia spinosa”) showed a high correlation between ascorbic acid content and the antioxidant activity (−0.89), while the correlation with the content of total polyphenols was lower (−0.45). For this group, our results can thus agree with Erdogan-Orhan and Kartal [15], for whom the polyphenolic fraction did not show a significant scavenging activity against DPPH, while it exerted a moderate scavenging effect against superoxide anion at 156 mg mL^−1^. However, our results are in disagreement with Ishiwata et al. [45], which found high correlation between the DPPH radical scavenging activity and polyphenols content. The antioxidant activity of varieties in the red group, on the contrary, showed a higher correlation with the content of total polyphenols (−0.67), respect to that with the content of ascorbic acid (−0.55), confirming, albeit partially, the influence that polyphenols can exert on the antioxidant activity. No group shows an appreciable correlation between antioxidant activity and β-carotene content.

### 3.5. Polyphenol Profile

Polyphenol profile is generally dependent on the genetic variation and the geographical conditions present at the time of the vegetable growth. Thus, a major number of information is welcome to identify the best varieties within a species that, for their quality and nutritional value can represent a given area. As far as we know, no studies are reporting the biochemical characterization of a so considerable number of varieties of apricot, traditional of the Campania regions, including polyphenols profile, obtained through UPLC. All data are shown in Table 2 and are indicated as μg g^−1^.

UPLC analysis -based on known molecules- recognized chlorogenic acid the most abundant phenolic acid. Its content ranged between 9.94 μg g^−1^ FW and 55.07 μg g^−1^ of fresh weight. High values of such molecule were observed in the cultivars “Baracca” and “Boccuccia di Eboli” (55.07 and 53.40 μg g^−1^ of fresh weight, respectively). The other three “Boccuccia” varieties, that is “Grossa”, “Liscia” and “Spinosa” showed just lower content of this molecule, in any case never inferior to 46.50 μg g^−1^ of fresh weight. Our data agree with Gottingerova et al. [46], which found the content of chlorogenic acid ranging between 0.69 and 21.94 mg 100 g^−1^ of fresh product. Caffeic acid ranged between 4.43 μg g^−1^ of fresh weight (found in the cultivar “Pollastrella”) and 38.77 μg g^−1^ (exhibited by cultivar “San Francesco”). Similarly, the content of gallic acid showed variability, ranging from 4.18 μg g^−1^ (in the variety “Signora”) to 11.70 μg g^−1^ of fresh weight (in the variety “Baracca”). *P*-coumaric and ferulic acids, the other two phenolic acids identified by UPLC did not exceed 6.34 μg g^−1^ and 11.07 μg g^−1^, respectively, both in the variety “Boccuccia grossa”. Catechin, epicatechin, and rutin resulted from the most predominant flavonoids recognized by UPLC-DAD, agreeing with Gundogdu et al. [47]. Catechin, in particular, resulted particularly abundant. Once again, “Baracca” and “Boccuccia di Eboli” showed the highest content of catechin (89.66 μg g^−1^ and 96.15 μg g^−1^, respectively); similarly, the other cultivar of the group “Boccuccia” exhibited a similar amount of the molecule, which ranged from 50.16 μg g^−1^ (“Boccuccia grossa”) to 52.14 μg g^−1^ (“Boccuccia spinosa”). “San Francesco” showed a high amount of caffeic acid and catechin, resulting from the cultivar with the highest content of epicatechin (49.03 μg g^−1^). The presence of a significant quantity of catechin and epicatechin in several of these apricot varieties represents an undoubted point of interest from human health. Catechins are recognized to decrease the hazard of some diseases. They can lead to the control of lipid and glucose/insulin metabolism [48]; they also have positive effects on the eye [49], nervous system, [50], and heart [51], and can exert anti-inflammatory effects [52] too. The molecule may also exhibit antibacterial and anti-biofilm activity [53,54]. Epicatechin and its metabolites may boost the muscle performance, act on the symptoms of cardiovascular and cerebrovascular diseases, and support human health, through actions of diabetes prevention and protection of the nervous system [55]. Rutin, which occurrence in the diet with vitamin C helps in the control of the fast blood glucose [56], was abundant in the “Boccuccia di Eboli” (53.76 μg g^−1^), “Scassulillo grande” (45.95 μg g^−1^), “San Francesco” (42.94 μg g^−1^), and “Baracca” (39.54 μg g^−1^). “B. grossa” and “B. spinosa” exhibited rutin content ranging between 19.92 μg g^−1^, and 24.76 μg g^−1^, respectively. The “Scassulillo” group of apricots, composed on “Scassulillo” and “Scassulillo grande” showed only as regard caffeic acid a similar trend. In all the other cases, the content of the singular polyphenols was higher in the cultivar “S. grande” than in “Scassulillo” Luteolin was detected only in traces and not in all cultivars. Quercetin and apigenin were usually undetected. They were present only in the cultivar “Baracca” and “Boccuccia di Eboli”; on the other hand, cultivar “Campana” showed traces also of quercetin. The almost complete absence of quercetin was once again in agreement with Iglesias-Carres et al., [57] and Gottingerova et al. [46], which detected this flavonoid only in one of the cultivars they analyzed (“Sefora”) and in traces. 

We have also considered all the available data (gallic acid, caffeic acid, chlorogenic acid, ferulic acid, *p*-coumaric acid, rutin, catechin, epicatechin, luteolin, ascorbic acid, β-carotene, total polyphenols, and antioxidant activity) and, after normalizing the data (in percentage, compared to the control “tipo 35”) we have performed a PCA (after data standardization). 

**Figure 2:**
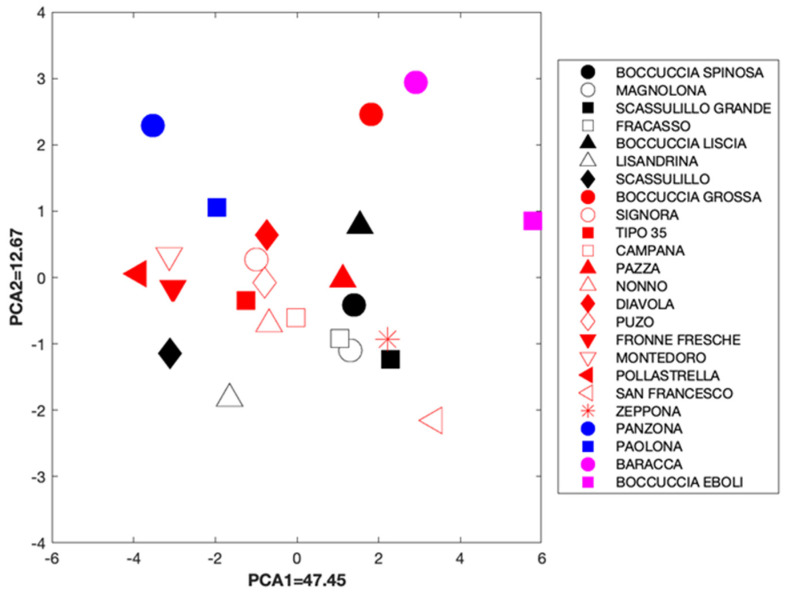
Varieties plotted using the first two PCA components and considering all the available (standardized) data.

Results (regarding the first two components) are shown in the Figure 2, where, for readability, we used separate symbols with a specific color (red, black, pink, and blue, full or empty) assigned to the varieties. In such a way, we represented each point with a symbol whose color is the group color to which the data point belongs (according to the dendrogram shown in Figure 1). Data points still can be considered as clustered according to the already introduced groups.

Thus, while taking into account that clustering may not be very evident, given, for example, the overlapping between the groups indicated by the colors red and black, the presence of two majority clusters could certainly be confirmed. The most abundant cluster was composed of 13 varieties (indicated by red colors, with full and empty symbols, including that “type 35” that was used as control); the second cluster comprised seven varieties (marked by full and empty black symbols). Two varieties, “Pazza” and “Zeppona”, indicated with red symbols, seemed very close to the black-symbolized “B. spinosa” and “S. grande” varieties. Two other pairs of varieties, “Panzona” and “Paolona” on one side, and “Baracca” and “Boccucia Eboli” on the other (indicated by solid blue and pink symbols, respectively), appeared to form two small groups apart. On the contrary, the “Magnolona” and “Fracasso” varieties were superimposable. From the clustering point of view, “Campana” and “Signora” varieties were also very close to each other too.

## 4. Conclusions

The valorization of fruit and vegetables representing a specific territory and community might be strategic to meet the key thoughts of the market and satisfy the growing need of the consumers to preserve their cultural traditions. Campania region represent one of the richest regions in Italy in terms of vegetal biodiversity, unfortunately not yet valorized in an adequate way, also outside its territory. Therefore, the Campania region is one of the most dynamic regions for the safeguard and valorization of the own vegetal genetic resource. As far as one can tell, this is the first time that a similar study regarded different traditional varieties of Campania apricots, grown and collected at the same conditions and in the same period, respectively, to counteract those variables capable of influencing their metabolic pathway. Our results suggest that such varieties can have great potential due to the content of phytochemicals useful for human health, compared to the control. The data obtained could contribute to the improvement of cultivation programs to safeguard the autochthonous biodiversity, considering consumers’ health. Finally, we should not forget that encouraging farmers to grow native fruit and vegetable varieties could also lead to an increase in their income.

## Figures and Tables

**Table 1:** Content of ascorbic acid, β-carotene, total polyphenols and antioxidant activity exhibited by the traditional varieties of apricots of the Campania region. Data represent the average (±SD) of three independent experiments. FW: fresh weight.

Varieties	Ascorbic Acid (mg 100 g^−1^ FW)	β-Carotene (mg 100 g^−1^ FW)	Total Polyphenols (mg 100 g^−1^ FW)	Antioxidant Activity (EC50, mg)
BARACCA	4.05 (±0.13)	0.408 (±0.52)	29.88 (±11.382)	10.15 (±0.04)
BOCCUCCIA EBOLI	6.04 (±0.09)	0.307 (±0.68)	35.87 (±15.29)	6.60 (±0.12)
BOCCUCCIA GROSSA	4.09 (±0.15)	0.223 (±0.26)	22.130 (±19.87)	12.79 (±0.16)
BOCCUCCIA LISCIA	7.73 (±0.36)	0.235 (±0.28)	22.38 (±12.57)	11.18 (±0.11)
BOCCUCCIA SPINOSA	9.80 (±0.21)	0.134 (±0.04)	22.020 (±9.32)	9.32 (±0.15)
CAMPANA	5.72 (±0.22)	0.134 (±0.06)	19.96 (±10.67)	7.46 (±0.07)
DIAVOLA	3.58 (±0.38)	0.126 (±0.03)	16.728 (±1.82)	11.49 (±0.10)
FRACASSO	9.27 (±0.21)	0.088 (±0.02)	20.168 (±8.43)	8.96 (±0.15)
FRONNE FRESCHE	4.07 (±0.10)	0.100 (±0.04)	9.952 (±3.31)	11.54 (±0.20)
LISANDRINA	8.79 (±0.10)	0.062 (±0.02)	12.821 (±2.21)	9.49 (±0.31)
MAGNOLONA	10.08 (±0.02)	0.152 (±0.04)	22.983 (±2.87)	8.41 (±0.03)
MONTEDORO	4.2 (±0.21)	0.06 (±0.02)	10.574 (±0.64)	14.87 (±0.47)
NONNO	5.24 (±0.16)	0.089 (±0.08)	17.173 (±2.90)	8.14 (±0.45)
PANZONA	3.56 (±0.35)	0.314 (±0.04)	9.142 (±0.92)	17.14 (±0.09)
PAOLONA	4.20 (±0.19)	0.271 (±0.07)	11.281 (±1.55)	12.27 (±0.15)
PAZZA	4.25 (±0.19)	0.147 (±0.03)	20.753 (±2.89)	10.12 (±0.33)
POLLASTRELLA	4.86 (±0.12)	0.134 (±0.05)	7.532 (±1.78)	13.49 (±0.25)
PUZO	3.59 (±0.20)	0.057 (±0.02)	17.433 (±2.86)	11.52 (±0.41)
SAN FRANCESCO	5.64 (±0.33)	0.092 (±0.02)	27.879 (±0.79)	6.88 (±0.22)
SCASSULILLO	8.92 (±0.17)	0.114 (±0.02)	8.303 (±2.65)	10.58 (±0.23)
SCASSULILLO GRANDE	9.85 (±0.24)	0.100 (±0.03)	24.424 (±3.87)	8.42 (±0.13)
SIGNORA	3.47 (±0.26)	0.208 (±0.03)	17.075 (±7.73)	9.26 (±0.10)
ZEPPONA	4.86 (±0.12)	0.112 (±0.05)	26.329 (±11.60)	7.62 (±0.08)
tipo 35	4.42 (±0.43)	0.194 (±0.03)	13.789 (±3.53)	8.98 (±0.23)

**Table 2:** Amount of polyphenols known present in the varieties of Campania apricots, detected by UPLC-DAD. Data are reported as μg g^−1^ of fresh product and are mean values ± SD of three determinations. QUE: quercetin; CAF: caffeic acid; GAL: gallic acid; CHLO: chlorogenic acid; CAT: catechin; EPIC:e picatechin; COUM: *p*-coumaric acid; RUT: rutin; FER: ferulic acid; LUT: luteolin; API: apigenin.

	QUE	CAF	GAL	CHLO	CAT	EPIC	COUM	RUT	FER	LUT	API
Baracca	0.286 (±0.03)	8.77 (±0.65)	11.70 (±0.91	55.07 (±4.24)	89.66 (±6.54)	36.34 (±3.27)	1.81 (±0.04)	39.54 (±3.74)	4.44 (±0.57)	0.00 (±0.00)	0.24 (±0.01)
Boccuccia Eboli	1.83 (±0.05)	27.16 (±1.67)	8.45 (±1.13)	53.40 (±2.67)	96.15 (±4.37)	40.88 (±3.33)	4.69 (±0.73)	53.76 (±4.57)	7.78 (±0.64)	4.75 (±0.21)	2.27 (±0.14)
Boccuccia Grossa	0.00 (±0.00)	13.97 (±1.13)	6.30 (±0.57)	46.50 (±4.67)	50.16 (±4.67)	28.09 (±1.57)	6.34 (±0.23)	19.92 (±1.23)	11.07 (±1.02)	2.70 (±0.97)	0.00 (±0.00)
Boccuccia Liscia	0.00 (±0.00)	20.26 (±1.57)	7.29 (±1.23)	48.12 (±2.67)	51.39 (±2.67)	30.31 (±3.09)	3.84 (±0.44)	20.86 (±1.13)	5.71 (±0.94)	2.14 (±0.07)	0.00 (±0.00)
Campana	2.82 (±0.03)	9.98 (±1.12)	5.34 (±1.07)	34.87 (±3.47)	55.64 (±3.35)	32.41 (±4.04)	1.93 (±0.06)	22.26 (±1.66)	2.78 (±0.67)	0.00 (±0.00)	0.00 (±0.00)
Diavola	0.00 (±0.00)	9.69 (±0.57)	7.71 (±1.44)	32.33 (±2.67)	42.88 (±1.57)	18.47 (±1.57)	2.64 (±023)	23.42 (±0.03)	3.36 (±0.03)	0.45 (±0.05)	0.00 (±0.00)
Fracasso	0.00 (±0.00)	15.80 (±0.57)	8.00 (±1.23)	38.18 (±2.12)	42.63 (±3.44)	11.17 (±0.97)	2.09 (±0.21)	42.89 (±1.67)	6.34 (±1.12)	2.99 (±0.45)	0.00 (±0.00)
Fronne Fresche	0.00 (±0.00)	2.46 (±0.43)	4.51 (±1.05)	20.09 (±1.54)	19.52 (±1.47)	7.19 (±1.02)	0.82 (±0.04)	26.19 (±2.01)	1.42 (±0.22)	2.39 (±0.37)	0.00 (±0.00)
Lisandrina	0.00 (±0.00)	11.08 (±0.84)	5.14 (±0.63)	14.24 (±1.02)	49.29 (±2.04)	12.57 (±1.02)	2.44 (±0.14)	9.62 (±0.67)	1.89 (±0.57)	2.69 (±0.08)	0.00 (±0.00)
Magnolona	0.00 (±0.00)	11.21 (±1.04)	7.02 (±0.97)	40.76 (±1.33)	51.17 (±2.04)	42.02 (±1.44)	2.80 (±0.73)	31.49 (±2.67)	2.30 (±0.13)	1.51 (±0.12)	0.00 (±0.00)
Montedoro	0.00 (±0.00)	10.50 (±1.34)	6.39 (±0.37)	29.97 (±2.67)	17.20 (±1.57)	8.06 (±1.36)	0.80 (±0.04)	15.47 (±1.03)	1.93 (±0.21)	0.00 (±0.00)	0.00 (±0.00)
Nonno	0.00 (±0.00)	9.83 (±0.57)	4.66 (±0.57)	42.55 (±1.15)	38.62 (±1.67)	19.61 (±1.33)	1.13 (±0.08)	24.15 (±1.05)	2.72 (±0.14)	3.25 (±0.45)	0.00 (±0.00)
Panzona	0.00 (±0.00)	2.86 (±0.44)	5.38 (±0.52)	17.01 (±1.23)	13.00 (±1.44)	16.93 (±1.06)	1.25 (±0.15)	19.19 (±0.81)	0.83 (±0.03)	1.07 (±0.02)	0.00 (±0.00)
Paolona	0.00 (±0.00)	7.96 (±0.70)	5.75 (±0.12)	22.12 (±1.44)	28.09 (±1.67)	8.30 (±0.74)	1.97 (±0.12)	25.16 (±1.67)	1.33 (±0.02)	3.51 (±0.43)	0.00 (±0.00)
Pazza	0.00 (±0.00)	10.59 (±0.77)	10.36 (±0.57)	14.73 (±0.67)	77.50 (±2.67)	16.68 (±1.23)	3.89 (±0.73)	32.77 (±1.52)	2.59 (±0.08)	5.91 (±0.57)	0.00 (±0.00)
Puzo	0.00 (±0.00)	12.84 (±0.62)	6.76 (±0.14)	33.29 (±2.23)	47.12 (±2.54)	21.36 (±1.57)	1.87 (±0.54)	17.76 (±1.24)	3.17 (±1.04)	2.50 (±0.15)	0.00 (±0.00)
San Francesco	0.00 (±0.00)	38.77 (±2.04)	7.24 (±1.07)	22.26 (±1.57)	65.55 (±2.06)	49.03 (±1.52)	4.83 (±1.04)	42.94 (±2.67)	1.77 (±0.12)	2.61 (±0.15)	0.00 (±0.00)
Scassulillo	0.00 (±0.00)	5.64 (±0.03)	5.83 (±0.03)	15.28 (±0.03)	24.04 (±0.03)	9.34 (±0.03)	1.16 (±0.03)	7.55 (±0.03)	1.26 (±0.03)	0.31 (±0.08)	0.00 (±0.03)
Scassulillo Grande	0.00 (±0.00)	12.28 (±0.81)	11.52 (±1.03)	32.61 (±0.45)	60.56 (±3.06)	38.76 (±2.67)	3.34 (±1.13)	45.95 (±2.55)	2.84 (±0.12)	0.00 (±0.00)	0.00 (±0.00)
Signora	0.00 (±0.00)	13.81 (±0.84)	4.18 (±0.12)	28.69 (±1.21)	54.92 (±3.67)	11.7 (±0.89)	1.61 (±0.04)	27.39 (±2.02)	1.76 (±0.02)	0.59 (±0.03)	0.00 (±0.00)
Zeppona	0.00 (±0.00)	20.43 (±1.67)	8.19 (±0.62)	30.09 (±2.14)	78.96 (±2.67)	48.02 (±1.57)	2.66 (±0.05)	30.32 (±2.05)	2.91 (±0.16)	1.93 (±0.02)	0.00 (±0.00)
Pollastrella	0.00 (±0.00)	4.43 (±0.57)	4.00 (±0.67)	9.94 (±1.01)	16.75 (±1.57)	13.73 (±1.23)	1.36 (±0.16)	12.55 (±1.15)	1.15 (±0.05)	0.00 (±0.00)	0.00 (±0.00)
Boccuccia Spinosa	0.00 (±0.00)	12.54 (±1.02)	7.03 (±0.21)	48.45 (±2.57)	52.14 (±2.23)	24.72 (±1.57)	2.17 (±0.04)	26.76 (±1.54)	6.94 (±0.32)	4.47 (±0.21)	0.00 (±0.00)
Tipo 35	0.00 (±0.00)	13.51 (±1.02)	5.32 (±0.87)	11.62 (±0.97)	39.92 (±2.05)	11.28 (±0.84)	1.74 (±0.04)	28.95 (±2.23)	2.43 (±0.04)	2.91 (±0.03)	0.00 (±0.00)
